# Mendelian randomization and Bayesian model averaging of autoimmune diseases and Long COVID

**DOI:** 10.3389/fgene.2024.1383162

**Published:** 2024-06-20

**Authors:** Jieni Feng, Jiankun Chen, Xiaoya Li, Xiaolei Ren, Junxu Chen, Zuming Li, Yuan Wu, Zhongde Zhang, Rongyuan Yang, Jiqiang Li, Yue Lu, Yuntao Liu

**Affiliations:** ^1^ The Second Clinical Medical College, Guangzhou University of Chinese Medicine, Guangzhou, China; ^2^ The Second Affiliated Hospital (Guangdong Provincial Hospital of Chinese Medicine), Guangzhou University of Chinese Medicine, Guangzhou, China; ^3^ Guangzhou Key Laboratory of Traditional Chinese Medicine for Prevention and Treatment of Emerging Infectious Diseases, Guangzhou, China; ^4^ The First Clinical Medical College, Guangzhou University of Chinese Medicine, Guangzhou, China; ^5^ State Key Laboratory of Traditional Chinese Medicine Syndrome, The Second Affiliated Hospital of Guangzhou University of Chinese Medicine, Guangzhou, China

**Keywords:** Long Covid, autoimmune diseases, Mendelian randomization, Bayesian model averaging, causality

## Abstract

**Background:**

Following COVID-19, reports suggest Long COVID and autoimmune diseases (AIDs) in infected individuals. However, bidirectional causal effects between Long COVID and AIDs, which may help to prevent diseases, have not been fully investigated.

**Methods:**

Summary-level data from genome-wide association studies (GWAS) of Long COVID (N = 52615) and AIDs including inflammatory bowel disease (IBD) (N = 377277), Crohn’s disease (CD) (N = 361508), ulcerative colitis (UC) (N = 376564), etc. were employed. Bidirectional causal effects were gauged between AIDs and Long COVID by exploiting Mendelian randomization (MR) and Bayesian model averaging (BMA).

**Results:**

The evidence of causal effects of IBD (OR = 1.06, 95% CI = 1.00–1.11, *p* = 3.13E-02), CD (OR = 1.10, 95% CI = 1.01–1.19, *p* = 2.21E-02) and UC (OR = 1.08, 95% CI = 1.03–1.13, *p* = 2.35E-03) on Long COVID was found. In MR-BMA, UC was estimated as the highest-ranked causal factor (MIP = 0.488, MACE = 0.035), followed by IBD and CD.

**Conclusion:**

This MR study found that IBD, CD and UC had causal effects on Long COVID, which suggests a necessity to screen high-risk populations.

## 1 Introduction

Autoimmune diseases (AIDs) are a group of diseases characterized by the body’s immune response to autologous antigens resulting in damage to its own tissues, affecting 5%–10% of the global population (Trier and Houen, 2023) and increasing in incidence (Lerner et al., 2015). More than 80 AIDs have been identified, including urticaria, asthma, psoriasis, Type 1 diabetes mellitus (T1D), rheumatoid arthritis (RA), inflammatory bowel disease (IBD) (including Crohn’s disease (CD) and ulcerative colitis (UC)), *etc.* AIDs can be divided into two subclasses: systemic and organ-specific (Marrack et al., 2001). Organ-specific AIDs produce an immune response only to the autoantigens of a specific organ, resulting in local damage (DiMeglio et al., 2018; Filippi et al., 2018), whereas systemic AIDs produce an immune response to a widely distributed autoantigen, resulting in widespread damage (Marrack et al., 2001; Rahman and Isenberg, 2008; Smolen et al., 2018; Ze et al., 2021). The presence of autoantibodies (autoAbs) is a common feature of AIDs and may increase a patient’s rate of infection with other diseases (Puel et al., 2022). It has been found that autoAbs are associated with the incidence rate and prognosis of the pandemic COVID-19 (Pablos et al., 2020; Sawalha et al., 2020; Knight et al., 2021) and may even be related to the emergence of the Long COVID (Su et al., 2022; Taghadosi et al., 2023). Long COVID refers to the persistence of symptoms beyond 3 months of SARS-CoV-2 infection that continue for no less than 2 months and aren’t caused by any other illness (Lechner-Scott et al., 2021; Mehandru and Merad, 2022; Raman et al., 2022).

Approximately 31%–69% of COVID-19 patients suffer from Long COVID (Groff et al., 2021), the pathogenesis of which is complex and hypotheses include (1) residual virus storage, (2) immune failure leading to slow clearance of the virus, (3) cross reactivity between SARS-CoV-2-specific antibodies and host proteins leading to autoimmunity, etc. (Ramakrishnan et al., 2021). There is still a large knowledge gap in the pathophysiology of Long COVID and a lack of targeted treatment (Ceban et al., 2022; Ceban et al., 2023). Therefore, finding the genetic association between AIDs and Long COVID may inspire the prevention and treatment of Long COVID. However, there is currently a lack of research on whether there is a causal relationship between the two or the direction of the causal relationship.

Mendelian randomization (MR) emerges as a more robust analytical approach for drawing reasonable etiological conclusions. MR leverages genetic variants as surrogates for the exposure, facilitating stronger causal inferences. MR offers a distinct advantage over other methods in terms of susceptibility to confounding factors (Wang et al., 2022). This is because germline genetic variations are assigned at random during meiosis, allowing them to serve as proxies for lifelong exposures without being influenced by the potentially biased effects of reverse causation. In addition, by extending the MR method, bidirectional MR has been employed for identifying the direction of causal effects between two interconnected traits (Sekula et al., 2016). MR-BMA is a multivariable MR approach that can be extended to the dimensions of high-throughput experiments and obtain risk factors from many candidate risk factors (Zuber et al., 2020). By reporting the model-averaged causal effect (MACE), MR-BMA can better and more consistently detect true risk factors for disease than either the inverse variance weighted (IVW) method or other variable selection methods (Zuber et al., 2020).

In this study, we utilized pooled statistics from genome-wide association studies (GWAS) for Long COVID and AIDs. The influence of several AIDs on Long COVID and the influence of Long COVID on AIDs were identified respectively and it was concluded that IBD, CD and UC have significant causal effects on Long COVID, which may be clinically helpful in identifying risk factors, screening high-risk groups and preventing disease progression.

## 2 Methods

### 2.1 Study design

**FIGURE 1:**
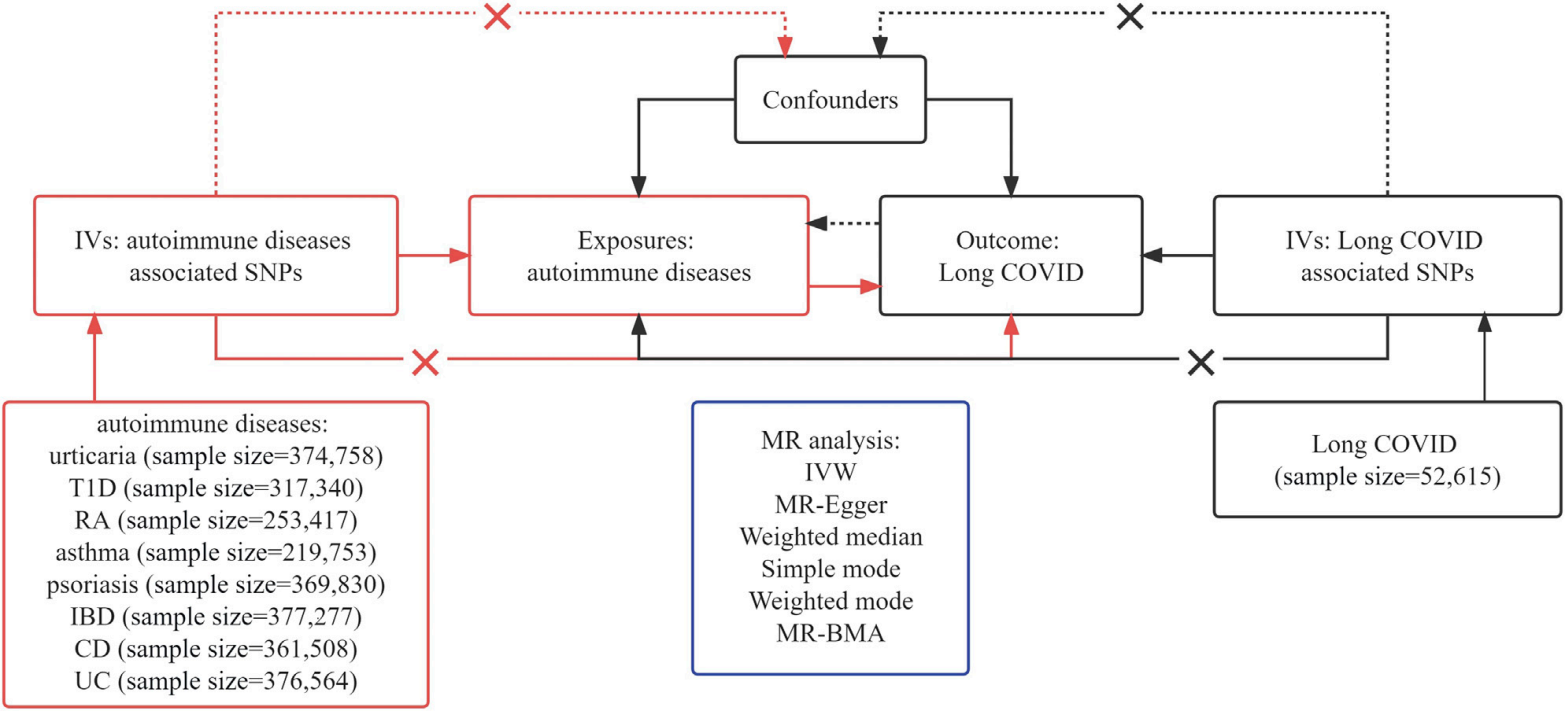
Assumptions and framework of the bidirectional Mendelian Randomization (MR) investigation on the links between autoimmune diseases (AIDs) and Long COVID.

Figure 1 presents a concise depiction of the bidirectional MR design. MR relies on three fundamental assumptions: 1) There is a strong and consistent correlation between the exposure and the chosen instrumental variable, representing the genetic variant. 2) There is no link between genetic variation and confounding factors. 3) Genetic variations influence outcomes exclusively via exposure, rather than alternate pathways (Kennedy et al., 2020). Our research employed summary-level data derived from publicly available GWAS conducted on Long COVID and AIDs. Our approach involved three distinct steps. Initially, we carefully identified genetic variants associated with AIDs to establish a causal inference from AIDs to Long COVID. Subsequently, we utilized genetic variants linked to Long COVID to infer the causality of Long COVID to AIDs. Finally, we performed MR-BMA for the positive results of univariable MR.

### 2.2 Genetic instrumental variables for AIDs and Long COVID

Summary statistics for AIDs were obtained from the FinnGen biobank analysis round 9 datasets (Table 1) (10,175 urticaria cases and 364,583 controls; 8,967 T1D cases and 308,373 controls; 12,555 RA cases and 240,862 controls; 9,631 asthma cases and 210,122 controls; 5,759 psoriasis cases and 364,071 controls; 7,625 IBD cases and 369,652 controls; 1,581 CD cases and 359,927 controls; 5,034 UC cases and 371,530 controls) (Kurki et al., 2023). Individuals of ambiguous sex, high genotypic deletion rate (>5%), high heterozygosity (+−4SD) and non-Finnish origin were excluded during the sample quality control step. Variants with high deletion rates (>2%), low Hardy–Weinberg Equilibrium *p*-values (<10^–6^), and low minor allele counts (<3) were excluded during the variant quality control step. To account for potential confounding factors, such as age, gender, ten principal components and genotype batch, the researchers at FinnGen employed mixed-model logistic regression techniques during their analyses (Wei et al., 2023). We want to acknowledge the participants and investigators of the FinnGen study.

**TABLE 1:** Summary statistics for AIDs.

Trait	Phenocode	Ncase	Ncontrol
urticaria	L12_URTICARIA	10175	364583
T1D	T1D_WIDE	8967	308373
RA	M13_RHEUMA	12555	240862
asthma	ALLERG_ASTHMA	9631	210122
psoriasis	L12_PSORI_VULG	5759	364071
IBD	K11_IBD_STRICT	7625	369652
CD	CHRONLARGE	1581	359927
UC	K11_UC_STRICT2	5034	371530

We acquired summary-level data for Long COVID from the most recently published GWAS available (Lammi et al., 2023), consisting of 6,407 cases (Long COVID after test-verified, self-reported or clinician-diagnosed SARS-CoV-2 infection) and 46,208 controls (individuals that had SARS-CoV-2 but did not develop Long COVID). Participants are defined as Long COVID cases when the criteria below are met. For studies with questionnaires, self-reported COVID-19 symptoms that cannot account for other diagnoses and reported continued significant influence on daily activities were adopted. For cohorts having electronic health records, diagnosis codes for Long COVID (Post COVID-19 condition, ICD-10 code U09 (.9)) were included. The study was reported in Nature’s news section (Ledford, 2023).

### 2.3 Determination of genetic instrumental variables

To adhere to the hypotheses outlined in Figure 1 of our study, we carefully selected single-nucleotide polymorphisms (SNPs) that fulfilled two important criteria: robustness and independence as predictors. This selection was based on the significant genome-wide results (*p* < 5 × 10^−8^) obtained from the published GWAS. Building upon existing research insights, we utilized SNP clumping techniques to identify distinct and independent loci. This involved establishing a linkage disequilibrium (LD) threshold of *r* ^2^ = 0.01 and implementing a clumping window of 5000 kb (Xie et al., 2023).

Rigorous data harmonization procedures were meticulously performed to ensure that the SNP’s impact on both the exposure and outcome aligned with the identical allele. In cases where SNPs exhibited various effect alleles from distinct strands, we diligently fixed the strand orientation to maintain consistency in effect alleles across datasets. Nevertheless, the harmonization of palindromic SNPs posed greater challenges since their alleles were identical on both strands. To mitigate any ambiguity in reporting the effect allele in the exposure and outcome GWAS, we decided to exclude these palindromic SNPs from the analysis (Bowden et al., 2017; Guo et al., 2023).

Finally, an assessment was conducted on the potency of each SNP using the F-statistics, an indicator dependent on the magnitude and accuracy of the genetic impact on the characteristic under consideration. SNPs displaying F-statistics below 10 were eliminated, as F-statistics exceeding 10 indicated adequate potency to uphold the credibility of the SNPs (Vaucher et al., 2018).

### 2.4 MR analysis

The primary method used to assess the relationship between AIDs and Long COVID was the IVW approach with multiplicative random effects. This method incorporates the Wald estimator of SNPs to estimate the effect. To ensure the validity of the IVW results, each SNP must satisfy the assumption of MR, specifically level-free pleiotropy. Since we used 8 exposures in the MR analysis, we applied a Bonferroni correction, meaning that a significance level of *p* < 0.00625 (0.05/8) was used. *p* values ranging from 0.00625 to 0.05 were regarded as suggestively significant.

Aside from the IVW method, several sensitivity analyses were employed as supplementary analysis methods, including MR-Egger and weighted median.

To examine the exclusion restriction assumption, we employed the MR-Egger regression intercept along with its corresponding 95% confidence intervals (CIs) to evaluate the extent of bias in causal estimates attributable to directional pleiotropy (Bowden et al., 2015). By employing MR-Egger analysis, we assessed the presence of pleiotropy in instrumental variables, wherein a nonzero intercept signifies potential bias in the IVW estimate (Burgess and Thompson, 2017). We employed heterogeneity indicators derived from the IVW approach (using a significance threshold of *p* < 0.05) to signify the presence of potential horizontal pleiotropy. Furthermore, the intercept derived from the MR-Egger regression served as an indicator for directional pleiotropy (where *p* < 0.05 indicated the presence of directional pleiotropy) (Vaucher et al., 2018). We used MRPRESSO to detect outliers and rerun the analysis after excluding them. The analyses were carried out using the TwoSampleMR package (version 0.5.7) (https://mrcieu.github.io/TwoSampleMR) and MRPRESSO (version 1.0) (https://github.com/rondolab/MR-PRESSO) within the R environment (version 4.2.1). We used Sangerbox Tools (Shen et al., 2022) for the figure.

### 2.5 MR-BMA

We utilized MR-BMA to further strengthen the reliability of our MR analysis and find out the best model for the causal effect of AIDs on Long COVID. Exposures that have causal effects on Long COVID were included in the model of the multivariate framework if they don’t result in multicollinearity and have a strong correlation with one of the model’s instrumental variables (IVs). Given there is a high degree of overlap in genetic risk factors between AIDs, we did not remove AIDs for multicollinearity to avoid deletion of important data. Posterior probability (PP) for every model and marginal probability of inclusion (MIP) for every risk factor were estimated to identify the best model. The direction and importance of the risk factors were determined using the MACE for each model.

Unlike MR (using MR-Egger and MRPRESSO for pleiotropic variants), MR-BMA allows pleiotropic effects (Zuber et al., 2020). Pleiotropic variants can be identified as outliers in the model fit in MR-BMA (Zuber et al., 2020). We used the Q-statistic and Cook’s distance for sensitivity analyses to detect outliers and influential variants. Any genetic variant with a Q-statistic>10 or a Cook’s distance > N exposure/N SNPs) is marked with the name of the variant in the diagnostic plots. After excluding them, we performed the analysis again. The analyses were carried out using the TwoSampleMR package (version 0.5.7) (https://mrcieu.github.io/TwoSampleMR) within the R environment (version 4.2.1). More details for MR-BMA can be found in previous studies (Zuber et al., 2020).

## 3 Results

### 3.1 Determination of genetic instrumental variables

We selected a total of 8 SNPs that were significantly associated with urticaria, 108 for T1D, 50 for RA, 23 for asthma, 36 for psoriasis, 62 for IBD, 7 for CD and 48 for UC, as listed in Supplementary Tables S1–S8. The F-statistics for these SNPs were greater than 100, indicating a strong association.

### 3.2 MR analysis

**FIGURE 2:**
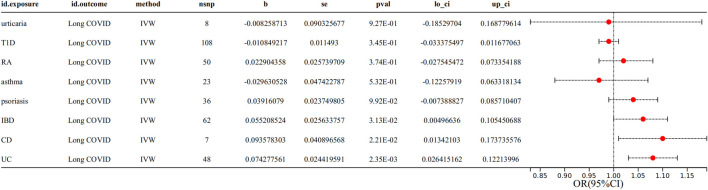
Associations between genetically predicted AIDs and Long COVID with SNPs.

Aiming to explore the causal impact of AIDs on Long COVID, MR analysis yielded the findings presented in Figure 2 and Supplementary Table S9. Genetically predicted UC showed a significant (*p* < 0.00625) association with the risk of Long COVID (IVW OR = 1.08, 95% CI = 1.03–1.13, *p* = 2.35E-03) (Supplementary Table S9). Genetically predicted IBD and CD showed a suggestively significant (0.00625 < *p* < 0.05) association with the risk of Long COVID (IBD IVW OR = 1.06, 95% CI = 1.00–1.11, *p* = 3.13E-02; CD IVW OR = 1.10, 95% CI = 1.01–1.19, *p* = 2.21E-02) (Supplementary Table S9).

The MR-Egger regression intercept term showed no clear pleiotropy among the SNPs in both datasets, with all *p*-values exceeding 0.05(Supplementary Table S9). No evidence of heterogeneity was observed in genetic variants associated with AIDs and Long COVID, as all *p*-values were above 0.05(Supplementary Table S9). The directions of the sensitivity analyses and the predicted impacts of the primary research were the same, including MR-Egger, weighted median, weighted mode and simple mode methods (Supplementary Table S9). The evidence presented demonstrated the stability of the outcomes derived from the IVW method. No outliers were detected with MRPRESSO.

However, there was no significant association observed between genetically predicted other AIDs (including urticaria, T1D, RA, asthma and psoriasis) and the risk of Long COVID. No evidence of horizontal pleiotropy or heterogeneity was observed, with all *p*-values exceeding 0.05(Supplementary Table S9). No outliers were detected with MRPRESSO.

There were no SNPs found to be genome-wide significantly associated with Long COVID. To ensure analytical accuracy, we opted not to use a relaxed *p*-value threshold.

### 3.3 MR-BMA

To ensure the reliability of the findings considering complicated genetic effects, MR-BMA analysis was employed for assessing the relationship of causality between Long COVID and AIDs. As genetically predicted IBD, CD and UC showed a significant association with the risk of Long COVID, we included IBD, CD and UC in the MR-BMA. We selected a total of 69 SNPs that were significantly associated with IBD, CD and UC (Supplementary Table S10).

UC was estimated as the highest-ranked causal factor (MIP = 0.488, MACE = 0.035), followed by IBD and CD (Table 2). UC was also the highest-ranked causal model (PP = 0.464), followed by IBD and CD (Table 3).

**TABLE 2:** AIDs ranked by their MIP.

Exposure	MIP	MACE
UC	0.488	0.035
IBD	0.423	0.028
CD	0.123	0.004

**TABLE 3:** Best individual AID models based on their PP.

Exposure	PP
UC	0.464
IBD	0.396
CD	0.106
IBD, UC	0.017
CD, IBD	0.01
CD, UC	0.007

**FIGURE 3:**
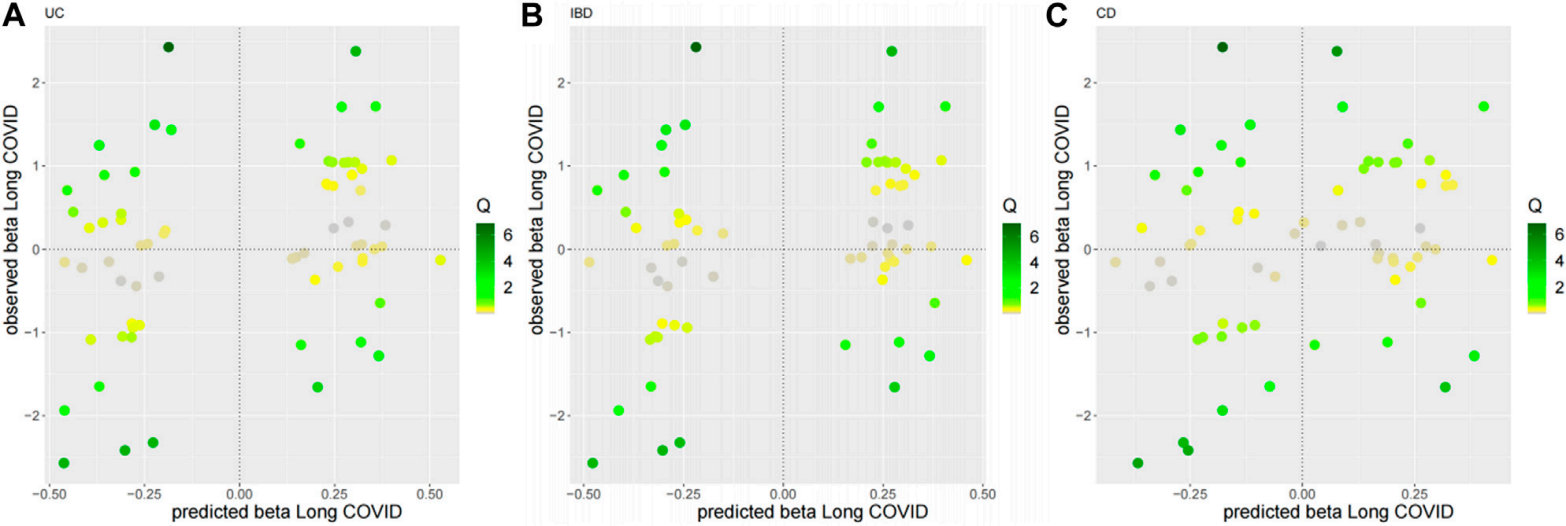
Diagnostic plots for outliers for the top three MR-BMA models. Any genetic variant with a Q-statistic>10 is marked with the name of the variant. **(A)** UC, **(B)** IBD, **(C)** CD.

**FIGURE 4:**
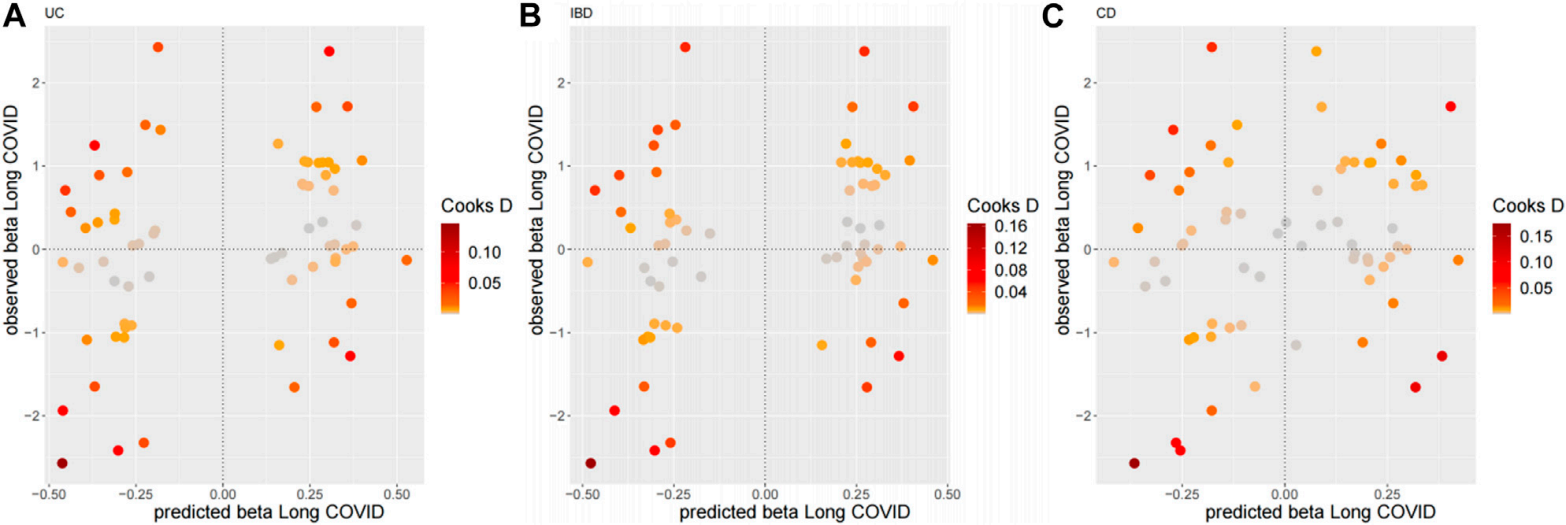
Diagnostic plots for influential genetic variants for the top three MR-BMA models. Predicted associations (*x*-axis) are plotted against observed associations (*y*-axis) for Long COVID. Any genetic variant with a Cook’s distance >0.04 (3/69) is marked with the name of the variant. **(A)** UC, **(B)** IBD, **(C)** CD.

As for the sensitivity analyses, any genetic variant with a Q-statistic>10 or a Cook’s distance >0.04 (3/69) is marked with the name of the variant. No outliers and influential variants were detected (Figures 3, 4).

## 4 Discussion

Based on GWAS data, our study applied a bidirectional MR analysis and MR-BMA to reveal the causal associations between AIDs and Long COVID. MR analysis indicated IBD, CD and UC may increase the risk of Long COVID, which was robust in different sensitivity analyses. MR-BMA further suggested that among the three AIDs, UC was most connected with Long COVID, followed by IBD and CD. In a longitudinal COVID-19 cohort study, AIDs were found to be significantly associated with the risk of Long COVID (Jacobs et al., 2023), which aligns with our findings. As far as we know, this is the first research focused on the causality between AIDs and Long COVID utilizing MR analysis and MR-BMA.

The presence of autoAbs is a common feature of AIDs (Damoiseaux et al., 2015). On one hand, during COVID-19 infection autoAbs may generate and cause AIDs (Liu et al., 2021; Sacchi et al., 2021). On the other hand, autoAbs can be physiological, as a transient tool to eliminate degraded self- and foreign antigen which causes no clinical disease (Avrameas and Selmi, 2013; Panda and Ding, 2015). The presence of autoAbs in COVID-19 patients correlates with increased antiviral humoral immune responses and inflammatory immune signature (Taeschler et al., 2022). Previous research shows that diverse functional autoAbs across a wide range of tissues and immunological and physiological functions were identified and validated in COVID-19 patients and that some of these autoAbs probably predated infection, whereas others were induced after infection (Davis et al., 2023). It has also shown the persistence of autoAbs in post-COVID individuals (Davis et al., 2023; Sin, 2023). There is a study demonstrating that autoAbs have an anti-correlation with anti-SARS-CoV-2 antibodies and are associated with distinct patterns of Long COVID (Su et al., 2022). In conclusion, the previous works mentioned above demonstrate that autoAbs generated before or after the infection of COVID-19, some of which are associated with AIDs, may lead to severe COVID-19 and Long COVID, suggesting a possibility that autoAbs associated with AIDs contribute to Long COVID.

Other factors such as immune cells and cytokines may also be crucial parts of the relationship between AIDs and Long COVID. A study of longitudinal clinical and autoantibody analyses found that a proinflammatory state was observed in patients with Long COVID featured by up or downregulation of various immune cells and cytokines, indicating a need to evaluate the role of immunomodulation in the treatment of Long COVID (Acosta-Ampudia et al., 2022). However, in general, research on whether autoAbs, immune cells and cytokines lead to Long COVID is still rarely seen. Until now researchers mainly focused on whether and how COVID-19 induces the production of autoAbs and the occurrence of AIDs but not on a reverse relation.

IBDs including CD and UC are AIDs. UC is limited to the colon and featured by mucosal inflammation. By contrast, CD, commonly associated with complications such as abscesses, fistulas and strictures, can cause transmural inflammation and affect any part of the gastrointestinal tract (Zhang and Li, 2014). AutoAbs such as anti-neutrophil cytoplasmic antibody (ANCA), anti-Saccharomyces cerevisiae antibody (ASCA), anti-intestinal goblet antibody (GBA) and anti-pancreatic antibody (PAB) exist in the serum of IBD patients (Fiocchi, 1998). ASCA is mainly seen in CD patients (Forcione et al., 2004), while ANCA is more common in UC patients and is used to distinguish UC and CD (Klebl et al., 2003). UC (Aydın and Taşdemir, 2021; Cayley, 2022; Xia et al., 2023) and CD (Senthamizhselvan et al., 2021; Tursi and Nenna, 2022) after COVID-19 have been reported. However, there is no other research indicating that UC, CD or ANCA, ASCA, GBA and PAB may serve as risk factors for Long COVID up to date. Immune cells and cytokines are also part of the relationship between COVID-19 and IBDs. For instance, a study found that interferon-γ (INF-γ) was the dominant driver of the expression of signaling lymphocytic activation molecule family member 7 (SLAMF7, CD319), whose engagement drove a strong wave of inflammatory cytokine expression in macrophages, and that SLAMF7-induced gene programs existed in gut macrophages from patients with active CD and in lung macrophages from patients with severe COVID-19 (Simmons et al., 2022). A cohort study found a significantly higher risk of COVID-19 related hospitalization and admission to intensive care in patients with CD and UC compared with background population with COVID-19 (Attauabi et al., 2022). A two-sample MR study found no evidence to support that IBDs increased the risk of COVID-19 susceptibility or severity (Ai and Yang, 2023). To our knowledge, our study is the first MR study working on the relationship between IBDs and Long COVID.

A study shows that Angiotensin-Converting Enzyme 2 (ACE2) serves as a receptor of SARS-CoV-2 (Hoffmann et al., 2020). Given that enhanced expressions of ACE2 in ileal and colonic tissue were observed in IBD (including CD and UC) (Garg et al., 2020), it is reasonable to draw a possible conclusion that IBD patients may suffer a higher risk of Long COVID mediated by ACE2.

IL-17, mainly produced by Th17 cells, plays an important role in IBD, specifically by affecting epithelial barrier function and innate immune response, promoting neutrophil recruitment and activation, and inducing the production of other pro-inflammatory cytokines and chemokines, thereby exacerbating intestinal inflammation (Moschen et al., 2019). Higher expression levels of IL-17 were observed in intestinal mucosa and peripheral blood of IBD patients compared with those of healthy controls (Sugihara et al., 2010; Song et al., 2013). As a large amount of IL-17 can be produced in cytokine storms induced by COVID-19 infection, some researchers regard that IL-17-dependent pathways may be common molecular mechanism between IBD and COVID-19 infection (Tao et al., 2022).

Some studies focused on the relationship between AIDs and COVID-19. However, a conclusion has not been reached. For instance, a study conducted in Hubei, China, indicated that patients with AIDs might be more susceptible to COVID-19 infection (Zhong et al., 2020). However, a study in northeast Italy suggested that in comparison with the general population, AIDs patients had a similar COVID-19 infection rate (Zen et al., 2020). A study in the United Kingdom showed that patients with severe AIDs had prominently higher mortality following COVID-19, but no differences were identified in the clinical endpoints among individuals with AIDs compared with those without AIDs who were hospitalized for COVID-19 during the initial surge of the pandemic (Arachchillage et al., 2022).

There are several advantages in our study based on GWAS summary-level data on a large scale. First, MR analysis overcame the issues of detecting confounders and reverse causality in typical observational and longitudinal research by generating practically unbiased causal estimates (Hemani et al., 2018). Second, the use of sensitivity analyses enabled us to further interrogate MR assumptions, which ensured notable changes in results could be found. Furthermore, the MR-BMA approach was used for identifying major causal AID while controlling for pleiotropy, which helped to evade the restrictions of linear regression-based MR approaches and strengthened our results’ robustness (Zuber et al., 2020). Effect estimates were given by MR-BMA biased towards the Null when a causal effect exists. However, MR-BMA trades bias for lessened variance, which means it can better and more stably detect true causal risk factors than either the conventional IVW method or other variable selection methods (Zuber et al., 2020).

There are limitations to consider in our MR study. First, the summary-level GWAS data utilized in this research is not stratified according to gender, age and disease severity, thus it was impossible to conduct a stratified analysis. Summary-level data also limits access to individual patient data which might provide richer insights. Second, our study heavily relies on data from specific populations (FinnGen for AIDs and the most recent GWAS for Long COVID), which may limit the external validity of the findings to other populations. Third, since the number of cases in the GWAS was relatively small, the analysis for Long COVID might be underpowered, leading to missing true associations or overestimating weak effects. IBD and CD have only suggestive significant causal effects on Long COVID. Although some sources of error in conventional observational cohort studies can be avoided, MR studies are affected by varying levels of influence from horizontal pleiotropy (Verbanck et al., 2018) and bias including measurement bias (Pierce and VanderWeele, 2012), weak instrumental variable bias (Burgess et al., 2011) and selection bias (Efron, 2011). The results need further validation in larger studies. Fourth, the analysis suggested that AIDs might increase the risk of Long COVID, but it is also possible that Long COVID could trigger or worsen AIDs. We were unable to explore the reverse causality (Long COVID triggering AIDs) in detail for lack of IVs. Also, the presence of autoAbs and immune response might be potential mechanisms linking AIDs to Long COVID as discussed above, with immune cells and cytokines likely playing a role in this relationship. Our study cannot definitively rule out these possibilities.

In conclusion, our MR study provides evidence of the causal effects of IBD, CD and UC respectively on Long COVID, which might be mediated by autoAbs, immune cells and cytokines. These results may shed fresh light on the bidirectional causal relationships between AIDs and Long COVID, especially attracting researchers’ and clinicians’ attention to the causal effect of AIDs on Long COVID but not only the reverse causal relation.

## Data Availability

The original contributions presented in the study are included in the article/Supplementary Material, further inquiries can be directed to the corresponding authors.

## References

[B1] Acosta-Ampudia Y. Monsalve D. M. Rojas M. Rodriguez Y. Zapata E. Ramirez-Santana C. (2022). Persistent autoimmune activation and proinflammatory state in post-Coronavirus disease 2019 syndrome. J. Infect. Dis. 225 (12), 2155–2162. 10.1093/infdis/jiac017 35079804 PMC8903340

[B2] Ai Q. Yang B. (2023). Are inflammatory bowel diseases associated with an increased risk of COVID-19 susceptibility and severity? A two-sample Mendelian randomization study. Front. Genet. 14, 1095050. 10.3389/fgene.2023.1095050 37152982 PMC10160392

[B3] Arachchillage D. J. Rajakaruna I. Pericleous C. Nicolson P. L. R. Makris M. Laffan M. (2022). Autoimmune disease and COVID-19: a multicentre observational study in the United Kingdom. Rheumatol. Oxf. 61 (12), 4643–4655. 10.1093/rheumatology/keac209 PMC8992350 35377457

[B4] Attauabi M. Dahlerup J. F. Poulsen A. Hansen M. R. Vester-Andersen M. K. Eraslan S. (2022). Outcomes and long-term effects of COVID-19 in patients with inflammatory bowel diseases - a Danish Prospective population-based cohort study with individual-level data. J. Crohns Colitis 16 (5), 757–767. 10.1093/ecco-jcc/jjab192 34755858 PMC8689957

[B5] Avrameas S. Selmi C. (2013). Natural autoantibodies in the physiology and pathophysiology of the immune system. J. Autoimmun. 41, 46–49. 10.1016/j.jaut.2013.01.006 23384670

[B6] Aydın M. F. Taşdemir H. (2021). Ulcerative colitis in a COVID-19 patient: a case report. Turk J. Gastroenterol. 32 (6), 543–547. 10.5152/tjg.2021.20851 34405821 PMC8975413

[B7] Bowden J. Davey Smith G. Burgess S. (2015). Mendelian randomization with invalid instruments: effect estimation and bias detection through Egger regression. Int. J. Epidemiol. 44 (2), 512–525. 10.1093/ije/dyv080 26050253 PMC4469799

[B8] Bowden J. Del Greco M. F. Minelli C. Davey Smith G. Sheehan N. Thompson J. (2017). A framework for the investigation of pleiotropy in two-sample summary data Mendelian randomization. Stat. Med. 36 (11), 1783–1802. 10.1002/sim.7221 28114746 PMC5434863

[B9] Burgess S. Thompson S. G. (2017). Interpreting findings from Mendelian randomization using the MR-Egger method. Eur. J. Epidemiol. 32 (5), 377–389. 10.1007/s10654-017-0255-x 28527048 PMC5506233

[B10] Burgess S. Thompson S. G. Collaboration C. C. G. (2011). Avoiding bias from weak instruments in Mendelian randomization studies. Int. J. Epidemiol. 40 (3), 755–764. 10.1093/ije/dyr036 21414999

[B11] Cayley W. E. Jr (2022). New-onset ulcerative colitis in patients with COVID-19. Am. Fam. Physician 106 (4), 362. 36260883

[B12] Ceban F. Kulzhabayeva D. Rodrigues N. B. Di Vincenzo J. D. Gill H. Subramaniapillai M. (2023). COVID-19 vaccination for the prevention and treatment of long COVID: a systematic review and meta-analysis. Brain Behav. Immun. 111, 211–229. 10.1016/j.bbi.2023.03.022 36990297 PMC10067136

[B13] Ceban F. Leber A. Jawad M. Y. Yu M. Lui L. M. W. Subramaniapillai M. (2022). Registered clinical trials investigating treatment of long COVID: a scoping review and recommendations for research. Infect. Dis. Lond. 54 (7), 467–477. 10.1080/23744235.2022.2043560 35282780 PMC8935463

[B14] Damoiseaux J. Andrade L. E. Fritzler M. J. Shoenfeld Y. (2015). Autoantibodies 2015: from diagnostic biomarkers toward prediction, prognosis and prevention. Autoimmun. Rev. 14 (6), 555–563. 10.1016/j.autrev.2015.01.017 25661979

[B15] Davis H. E. McCorkell L. Vogel J. M. Topol E. J. (2023). Long COVID: major findings, mechanisms and recommendations. Nat. Rev. Microbiol. 21 (3), 133–146. 10.1038/s41579-022-00846-2 36639608 PMC9839201

[B16] DiMeglio L. A. Evans-Molina C. Oram RAJTL (2018). Type 1 diabetes. Lancet 391 (10138), 2449–2462. 10.1016/S0140-6736(18)31320-5 29916386 PMC6661119

[B17] Efron B. (2011). Tweedie's Formula and selection bias. J. Am. Stat. Assoc. 106 (496), 1602–1614. 10.1198/jasa.2011.tm11181 22505788 PMC3325056

[B18] Filippi M. Bar-Or A. Piehl F. Preziosa P. Solari A. Vukusic S. (2018). Multiple sclerosis. Nat. Rev. Dis. Prim. 4 (1), 43. 10.1038/s41572-018-0041-4 30410033

[B19] Fiocchi C. (1998). Inflammatory bowel disease: etiology and pathogenesis. Gastroenterology 115 (1), 182–205. 10.1016/s0016-5085(98)70381-6 9649475

[B20] Forcione D. G. Rosen M. J. Kisiel J. B. Sands B. E. (2004). Anti-Saccharomyces cerevisiae antibody (ASCA) positivity is associated with increased risk for early surgery in Crohn's disease. Gut 53 (8), 1117–1122. 10.1136/gut.2003.030734 15247177 PMC1774147

[B21] Garg M. Royce S. G. Tikellis C. Shallue C. Batu D. Velkoska E. (2020). Imbalance of the renin-angiotensin system may contribute to inflammation and fibrosis in IBD: a novel therapeutic target? Gut 69 (5), 841–851. 10.1136/gutjnl-2019-318512 31409604

[B22] Groff D. Sun A. Ssentongo A. E. Ba D. M. Parsons N. Poudel G. R. (2021). Short-term and long-term rates of Postacute sequelae of SARS-CoV-2 infection: a systematic review. JAMA Netw. Open 4 (10), e2128568. 10.1001/jamanetworkopen.2021.28568 34643720 PMC8515212

[B23] Guo Y. Li D. Hu Y. (2023). Appraising the associations between systemic Iron Status and Epigenetic Clocks: a genetic correlation and bidirectional Mendelian randomization study. Am. J. Clin. Nutr. 118 (1), 41–49. 10.1016/j.ajcnut.2023.05.004 37146762

[B24] Hemani G. Zheng J. Elsworth B. Wade K. H. Haberland V. Baird D. (2018). The MR-Base platform supports systematic causal inference across the human phenome. Elife 7, e34408. 10.7554/eLife.34408 29846171 PMC5976434

[B25] Hoffmann M. Kleine-Weber H. Schroeder S. Kruger N. Herrler T. Erichsen S. (2020). SARS-CoV-2 cell Entry Depends on ACE2 and TMPRSS2 and is Blocked by a clinically proven Protease Inhibitor. Cell 181 (2), 271–280. 10.1016/j.cell.2020.02.052 32142651 PMC7102627

[B26] Jacobs E. T. Catalfamo C. J. Colombo P. M. Khan S. M. Austhof E. Cordova-Marks F. (2023). Pre-existing conditions associated with post-acute sequelae of COVID-19. J. Autoimmun. 135, 102991. 10.1016/j.jaut.2022.102991 36634460 PMC9816074

[B27] Kennedy O. J. Pirastu N. Poole R. Fallowfield J. A. Hayes P. C. Grzeszkowiak E. J. (2020). Coffee Consumption and Kidney function: a Mendelian randomization study. Am. J. Kidney Dis. 75 (5), 753–761. 10.1053/j.ajkd.2019.08.025 31837886

[B28] Klebl F. H. Bataille F. Bertea C. R. Herfarth H. Hofstadter F. Scholmerich J. (2003). Association of perinuclear antineutrophil cytoplasmic antibodies and anti-Saccharomyces cerevisiae antibodies with Vienna classification subtypes of Crohn's disease. Inflamm. Bowel Dis. 9 (5), 302–307. 10.1097/00054725-200309000-00003 14555913

[B29] Knight J. S. Caricchio R. Casanova J. L. Combes A. J. Diamond B. Fox S. E. (2021). The intersection of COVID-19 and autoimmunity. J. Clin. Invest 131 (24), e154886. 10.1172/JCI154886 34710063 PMC8670833

[B30] Kurki M. I. Karjalainen J. Palta P. Sipilä T. P. Kristiansson K. Donner K. M. (2023). FinnGen provides genetic insights from a well-phenotyped isolated population. Nature 613 (7944), 508–518. 10.1038/s41586-022-05473-8 36653562 PMC9849126

[B31] Lammi V. Nakanishi T. Jones S. E. Andrews S. J. Karjalainen J. Cortés B. Genome-wide association study of long COVID. 2023:2023. medRxiv06.29.23292056. 10.1038/s41588-025-02100-w PMC12165857 40399555

[B32] Lechner-Scott J. Levy M. Hawkes C. Yeh A. Giovannoni G. (2021). Long COVID or post COVID-19 syndrome. Mult. Scler. Relat. Disord. 55, 103268. 10.1016/j.msard.2021.103268 34601388 PMC8447548

[B33] Ledford H. (2023). Gene linked to long COVID found in analysis of thousands of patients. Nature 619 (7970), 445. 10.1038/d41586-023-02269-2 37433943

[B34] Lerner A. Jeremias P. Matthias T. (2015). The World incidence and Prevalence of autoimmune diseases is increasing. Int. J. Celiac Dis. 3 (4), 151–155. 10.12691/ijcd-3-4-8

[B35] Liu Y. Sawalha A. H. Lu Q. (2021). COVID-19 and autoimmune diseases. Curr. Opin. Rheumatology 33 (2), 155–162. 10.1097/bor.0000000000000776 PMC7880581 33332890

[B36] Marrack P. Kappler J. Kotzin B. L. (2001). Autoimmune disease: why and where it occurs. Nat. Med. 7 (8), 899–905. 10.1038/90935 11479621

[B37] Mehandru S. Merad M. (2022). Pathological sequelae of long-haul COVID. Nat. Immunol. 23 (2), 194–202. 10.1038/s41590-021-01104-y 35105985 PMC9127978

[B38] Moschen A. R. Tilg H. Raine T. (2019). IL-12, IL-23 and IL-17 in IBD: immunobiology and therapeutic targeting. Nat. Rev. Gastroenterol. Hepatol. 16 (3), 185–196. 10.1038/s41575-018-0084-8 30478416

[B39] Pablos J. L. Galindo M. Carmona L. Lledo A. Retuerto M. Blanco R. (2020). Clinical outcomes of hospitalised patients with COVID-19 and chronic inflammatory and autoimmune rheumatic diseases: a multicentric matched cohort study. Ann. Rheum. Dis. 79 (12), 1544–1549. 10.1136/annrheumdis-2020-218296 32796045

[B40] Panda S. Ding J. L. (2015). Natural antibodies bridge innate and adaptive immunity. J. Immunol. 194 (1), 13–20. 10.4049/jimmunol.1400844 25527792

[B41] Pierce B. L. VanderWeele T. J. (2012). The effect of non-differential measurement error on bias, precision and power in Mendelian randomization studies. Int. J. Epidemiol. 41 (5), 1383–1393. 10.1093/ije/dys141 23045203

[B42] Puel A. Bastard P. Bustamante J. Casanova J. L. (2022). Human autoantibodies underlying infectious diseases. J. Exp. Med. 219 (4), e20211387. 10.1084/jem.20211387 35319722 PMC8952682

[B43] Rahman A. Isenberg D. A. (2008). Systemic lupus Erythematosus. N. Engl. J. Med. 358 (9), 929–939. 10.1056/NEJMra071297 18305268

[B44] Ramakrishnan R. K. Kashour T. Hamid Q. Halwani R. Tleyjeh I. M. (2021). Unraveling the Mystery Surrounding post-acute sequelae of COVID-19. Front. Immunol. 12, 686029. 10.3389/fimmu.2021.686029 34276671 PMC8278217

[B45] Raman B. Bluemke D. A. Luscher T. F. Neubauer S. (2022). Long COVID: post-acute sequelae of COVID-19 with a cardiovascular focus. Eur. Heart J. 43 (11), 1157–1172. 10.1093/eurheartj/ehac031 35176758 PMC8903393

[B46] Sacchi M. C. Tamiazzo S. Stobbione P. Agatea L. De Gaspari P. Stecca A. (2021). SARS-CoV-2 infection as a trigger of autoimmune response. Clin. Transl. Sci. 14 (3), 898–907. 10.1111/cts.12953 33306235 PMC8212749

[B47] Sawalha A. H. Zhao M. Coit P. Lu Q. (2020). Epigenetic dysregulation of ACE2 and interferon-regulated genes might suggest increased COVID-19 susceptibility and severity in lupus patients. Clin. Immunol. 215, 2020.03.30.20047852. 10.1101/2020.03.30.20047852 PMC7139239 32276140

[B48] Sekula P. Del Greco M. F. Pattaro C. Kottgen A. (2016). Mendelian randomization as an approach to assess causality using observational data. J. Am. Soc. Nephrol. 27 (11), 3253–3265. 10.1681/ASN.2016010098 27486138 PMC5084898

[B49] Senthamizhselvan K. Ramalingam R. Mohan P. Kavadichanda C. Badhe B. Hamide A. (2021). *De novo* Crohn's disease triggered after COVID-19: is COVID-19 more Tha n an infectious disease? ACG Case Rep. J. 8 (8), e00652. 10.14309/crj.0000000000000652 34476279 PMC8386903

[B50] Shen W. Song Z. Zhong X. Huang M. Shen D. Gao P. (2022). Sangerbox: a comprehensive, interaction-friendly clinical bioinformatics analysis platform. iMeta 1 (3), e36. 10.1002/imt2.36 38868713 PMC10989974

[B51] Simmons D. P. Nguyen H. N. Gomez-Rivas E. Jeong Y. Jonsson A. H. Chen A. F. (2022). SLAMF7 engagement superactivates macrophages in acute and chronic inflammation. Sci. Immunol. 7 (68), eabf2846. 10.1126/sciimmunol.abf2846 35148199 PMC8991457

[B52] Sin D. D. (2023). Is long COVID an autoimmune disease? Eur. Respir. J. 61 (1), 2202272. 10.1183/13993003.02272-2022 36634924

[B53] Smolen J. S. Aletaha D. Barton A. Burmester G. R. Emery P. Firestein G. S. (2018). Rheumatoid arthritis. Nat. Rev. Dis. Prim. 4 (1), 18001. 10.1038/nrdp.2018.1 29417936

[B54] Song L. Zhou R. Huang S. Zhou F. Xu S. Wang W. (2013). High intestinal and systemic levels of interleukin-23/T-helper 17 pathway in Chinese patients with inflammatory bowel disease. Mediat. Inflamm. 2013, 425915. 10.1155/2013/425915 PMC3870108 24382939

[B55] Su Y. Yuan D. Chen D. G. Ng R. H. Wang K. Choi J. (2022). Multiple early factors anticipate post-acute COVID-19 sequelae. Cell 185 (5), 881–895.e20. 10.1016/j.cell.2022.01.014 35216672 PMC8786632

[B56] Sugihara T. Kobori A. Imaeda H. Tsujikawa T. Amagase K. Takeuchi K. (2010). The increased mucosal mRNA expressions of complement C3 and interleukin-17 in inflammatory bowel disease. Clin. Exp. Immunol. 160 (3), 386–393. 10.1111/j.1365-2249.2010.04093.x 20089077 PMC2883109

[B57] Taeschler P. Cervia C. Zurbuchen Y. Hasler S. Pou C. Tan Z. (2022). Autoantibodies in COVID-19 correlate with antiviral humoral responses and distinct immune signatures. Allergy 77 (8), 2415–2430. 10.1111/all.15302 35364615 PMC9111424

[B58] Taghadosi M. Safarzadeh E. Asgarzadeh A. Roghani S. A. Shamsi A. Jalili C. (2023). Partners in crime: autoantibodies complicit in COVID-19 pathogenesis. Rev. Med. Virol. 33 (2), e2412. 10.1002/rmv.2412 36471421 PMC9877745

[B59] Tao S. S. Wang X. Y. Yang X. K. Liu Y. C. Fu Z. Y. Zhang L. Z. (2022). COVID-19 and inflammatory bowel disease crosstalk: from emerging association to clinical proposal. J. Med. Virol. 94 (12), 5640–5652. 10.1002/jmv.28067 35971954 PMC9538900

[B60] Trier N. H. Houen G. (2023). Antibody cross-reactivity in Auto-immune diseases. Int. J. Mol. Sci. 24 (17), 13609. 10.3390/ijms241713609 37686415 PMC10487534

[B61] Tursi A. Nenna R. (2022). COVID-19 as a trigger for *de novo* Crohn's disease. Inflamm. bowel Dis. 28 (6), e76–e77. 10.1093/ibd/izab298 35657373 PMC8690164

[B62] Vaucher J. Keating B. J. Lasserre A. M. Gan W. Lyall D. M. Ward J. (2018). Cannabis use and risk of schizophrenia: a Mendelian randomization study. Mol. Psychiatry 23 (5), 1287–1292. 10.1038/mp.2016.252 28115737 PMC5984096

[B63] Verbanck M. Chen C. Y. Neale B. Do R. (2018). Detection of widespread horizontal pleiotropy in causal relationships inferred from Mendelian randomization between complex traits and diseases. Nat. Genet. 50 (5), 693–698. 10.1038/s41588-018-0099-7 29686387 PMC6083837

[B64] Wang Q. Shi Q. Lu J. Wang Z. Hou J. (2022). Causal relationships between inflammatory factors and multiple myeloma: a bidirectional Mendelian randomization study. Int. J. Cancer 151 (10), 1750–1759. 10.1002/ijc.34214 35841389

[B65] Wei Y. Lu X. Liu C. (2023). Gut microbiota and chronic obstructive pulmonary disease: a Mendelian randomization study. Front. Microbiol. 14, 1196751. 10.3389/fmicb.2023.1196751 37405157 PMC10315658

[B66] Xia C. Dissanayake J. Badov D. (2023). A new Onset of ulcerative colitis post-COVID-19: a case report. Cureus 15 (3), e36257. 10.7759/cureus.36257 37069864 PMC10105639

[B67] Xie J. Huang H. Liu Z. Li Y. Yu C. Xu L. (2023). The associations between modifiable risk factors and nonalcoholic fatty liver disease: a comprehensive Mendelian randomization study. Hepatology 77 (3), 949–964. 10.1002/hep.32728 35971878

[B68] Ze X. X. Joseph S. M. Song Guo Z. (2021). An updated advance of autoantibodies in autoimmune diseases. Autoimmun. Rev. 20 (2), 102743. 10.1016/j.autrev.2020.102743 33333232

[B69] Zen M. Fuzzi E. Astorri D. Saccon F. Padoan R. Ienna L. (2020). SARS-CoV-2 infection in patients with autoimmune rheumatic diseases in northeast Italy: a cross-sectional study on 916 patients. J. Autoimmun. 112, 102502. 10.1016/j.jaut.2020.102502 32527675 PMC7832807

[B70] Zhang Y. Z. Li Y. Y. (2014). Inflammatory bowel disease: pathogenesis. World J. Gastroenterol. 20 (1), 91–99. 10.3748/wjg.v20.i1.91 24415861 PMC3886036

[B71] Zhong J. Shen G. Yang H. Huang A. Chen X. Dong L. (2020). COVID-19 in patients with rheumatic disease in Hubei province, China: a multicentre retrospective observational study. Lancet Rheumatol. 2 (9), e557–e564. 10.1016/S2665-9913(20)30227-7 32838309 PMC7333992

[B72] Zuber V. Colijn J. M. Klaver C. Burgess S. (2020). Selecting likely causal risk factors from high-throughput experiments using multivariable Mendelian randomization. Nat. Commun. 11 (1), 29. 10.1038/s41467-019-13870-3 31911605 PMC6946691

